# The need for dedicated time for medical physicists practice quality improvement efforts in radiation oncology department: A commentary

**DOI:** 10.1002/acm2.13515

**Published:** 2022-01-18

**Authors:** Richard Zellars, Christopher Njeh, Scott Marquette

**Affiliations:** ^1^ Department of Radiation Oncology, Indiana University, School of Medicine Indianapolis Indiana USA

## 1 INTRODUCTION

There is no universally accepted definition of quality improvement (QI). However, the American Board of Radiology (ABR) defines “QI” as “a systematic approach to the study of healthcare and/or a commitment to efforts to continuously improve performance and outcomes in healthcare”. According to Kruskal et al.,^[^ ^1^ ^]^ QI in radiation oncology includes “(a) quality assurance programs for continuous improvements in quality, (b) processes to improve staff and patient safety, and (c) procedures to improve the clinical, technical, and therapy performance of all staff”.^[^ ^1^ ^]^ Fundamentally, QI techniques are, well founded methods to drive change and improve efficiency. The goal of QI is therefore to create practical processes and structures that will introduce positive change into a work environment in a reproducible and sustainable way that is non‐disruptive and at an acceptable cost. There are many forces that can drive the creation of QI programs in radiation oncology. The first is the desire to provide high‐quality patient care, which is defined by the Institute of Medicine as “safe, effective, patient‐centered, timely, efficient, and equitable care”.^[^ ^2^ ^]^ The second is the mandate of accrediting bodies such as the Joint Commission and the American College of Radiology (ACR), whose accrediting standards further support this goal. The third is the economic incentives to provide high‐quality care at an affordable cost.^[^ ^3^ ^]^

Clinical medical physicists (MPs) are often viewed as the custodians of quality in radiation therapy department. Radiation therapy is a long‐complicated process and therefore has numerous avenues for potential QI endeavors.^[^ ^4^, ^5^ ^]^ These QI initiatives demand time and resources to be successful. More often, when time is not reserved, these initiatives become administrative burdens on the staff adding to their already established workflow. To make QI relevant, feasible and sustainable, it is necessary to embed it into MP workflow. This act transforms QI from a burden, which places an extra demand on physicists’ time, into an exercise of team ingenuity. Clinical MPs dedicated time is therefore recommended to support QI. The justifications for this recommendation are presented in this commentary.

## 2 NEED FOR MPs’ QI

### 2.1 Accreditation requirements

Hospital accreditation is an external systematic assessment of a hospital's structures, processes, and results by an independent professional body using pre‐established accepted optimum standards. Accreditation has an important role in establishing standards and in improving the quality, safety, effectiveness, and efficiency of hospital services.^[^ ^6^ ^]^ There are three professional organizations that may provide radiation oncology accreditation: the American College of Radiology (ACR), the American Society for Radiation Oncology (ASTRO), and the American College of Radiation Oncology (ACRO).^[^ ^7^, ^8^, ^9^ ^]^ The accreditation programs from ACR, ASTRO, and ACRO are Radiation Oncology Practice Accreditation (ROPA), Accreditation Programs for Excellence (APEX), and Practice Accreditation Program (PAP), respectively. These programs provide radiation oncologists with an independent and impartial peer review. Facility staff, equipment, treatment‐planning, treatment records, patient‐safety policies, and quality control/quality assessment activities are all assessed.^[^ ^7^, ^8^, ^9^ ^]^ ACR established ROPA in 1986. ACRO's PAP was initiated in 1996 as a service to ACRO members.^[^ ^7^ ^]^ ASTRO unveiled its accreditation program for excellence in late 2015.

### 2.2 Maintenance of Certification

The American Board of Medical Specialties (ABMS) – a 24‐member board, representing all medical subspecialties in the USA, in March 2000, agreed to initiate specialty‐specific Maintenance of Certification (MOC) programs. Diplomats are no longer issued lifetime certification, instead they need to provide documentations of continued learning and QI. MOC recognizes that in addition to medical knowledge, several essential elements such as communication skills involved in the delivering quality care must be developed and maintained throughout one's career.^[^ ^10^, ^11^ ^]^

The ABR representing diagnostic radiology, radiation oncology, and radiation physics has developed their own MOC program, which was approved by the ABMS, and initiated with full implementation for all three disciplines starting in 2007.^[^ ^10^, ^11^ ^]^ The ABR MOC has four components: professional standing, lifelong learning and self‐assessment, cognitive expertise, and evaluation of practice performance. The self‐evaluation of practice performance includes the process of continuing QI and is entitled “Practice Quality Improvement” (PQI).^[^ ^12^ ^]^ The ABR's guidelines state that “every radiologic physics diplomate must complete a PQI project. The choice of PQI activities and projects are to meet the spirit of the definition of QI”.^[^ ^10^, ^14^ ^]^ Medical Physics embodies a wide range of clinical aspects and ABR has identified five possible PQI areas: safety of patients, employees and public, accuracy of analysis and calculation, report turnaround and communication issues, and practice guidelines and standards and surveys.^[^ ^10^ ^]^ In addition, the diplomate must demonstrate a commitment to maintaining competency as a radiologic physicist.^[^ ^10^ ^]^ Effective from 15 March 2016, the ABR instituted the continuous certification and annual “look‐back” processes which in part requires diplomates to have completed at least one PQI project in the previous 3 years. In a Medical Physics point/counter point article, Njeh et al.^[^ ^15^ ^]^ argued that PQI project could provide background material for research and publication.

## 3 MEDICAL PHYSICISTS’ PQI

Methodical PQI has never been a mainstream MPs’ activity, but the forgone sections have established the need for MPs to be active participants in these projects. This has been echoed in the growing request for PQI training to be included in the medical physicist residence syllabus.^[^ ^16^ ^]^ The benefits to patients, clinicians, and healthcare providers of engaging in PQI are considerable, but there are many challenges involved in designing, delivering, and sustaining a QI intervention.^[^ ^17^ ^]^ However, to have successful PQI projects some of these challenges need to be addressed: training, leadership support, time allocation, appropriate tools, mechanism for data collection, financial resources, human resources, and selecting the right project.^[^ ^13^, ^17^ ^]^

### 3.1 Training

MP needs to be trained in the process and procedures of PQI as they affect an individual's practice of radiologic Physics.^[^ ^10^ ^]^ This education requirement is echoed by Medical Physics residency programs accrediting agencies. Nonetheless, recent surveys indicate that most programs lack a formal program to support this learning.^[^ ^16^, ^18^ ^]^ The success of a PQI project depends on proper training on effective use of QI methodology.^[^ ^13^ ^]^ Six Sigma and Lean are more common QI methods.^[^ ^5^, ^19^ ^]^ Six Sigma reduce process variation by decreasing defects to a specific statistical measure. Six Sigma projects use a five‐phased process known as DMAIC (define, measure, analyze, improve, and control).^[^ ^20^ ^]^ The main emphasis of Lean is on cutting out unnecessary and wasteful steps in the delivery of a service. Lean uses a technique called value stream mapping. The next step is to apply the 5S (sort, simplify, sweep, standardize, and self‐discipline).^[^ ^21^, ^22^, ^23^ ^]^ Six Sigma and Lean have a complementary relationship with each other and can be combined as Lean Six Sigma. The synergetic adoption of these methods allows the creation of a continuous process flow that eliminates waste (Lean) and reduces process variation (Six Sigma), to achieve and maintain the best quality.^[^ ^24^ ^]^

Education and training are also required in other quality control tools like root cause analysis (RCA), failure mode and effect analysis (FMEA),^[^ ^25^ ^]^ and incident reporting and learning (IRL). RCA is a reactive retrospective approach used to ascertain the “root cause” of a problem that has already occurred, whereas FMEA is a proactive prospective systematic approach that is used to identify and understand causes, contributing factors, and effects of potential failures on a process, system, or practice. IRL is about using the opportunities from reported actual or potential incidents and analyzing them to determine the systemic and human factors involved.^[^ ^22^, ^26^, ^27^ ^]^ IRL is a reactive and retrospective look at a known error.

### 3.2 Leadership support

Critical elements in any quality program include leadership willingness to experiment and take risks. Institutional leadership and support send the message that all quality‐related efforts are valued and constitute a central component of the institution's mission. This important message should be enhanced by tangible support. This support may be financial such as the provision of human resources such as a departmental quality coordinator, or administrative, such as establishing and facilitating interdepartmental quality forums or adverse event reporting systems. Further leadership support can be demonstrated by the acknowledgment of efforts and successes.^[^ ^1^, ^13^, ^28^, ^29^, ^30^ ^]^

Leadership support is more critical when there is a bump in the road. As Hawkins^[^ ^31^ ^]^ eloquently states “There will be moments during all performance improvement projects when things do not go as planned or unforeseeable obstacles arise. If there is not a buy‐in from leadership—from people to whom members of your department look for guidance—then the initiative will fail. Simply engaging these individuals is likely not enough. As a QI project leader, you must clearly show key leadership stakeholders why the desired change is necessary, and how you plan to achieve the desired results. Hopefully, they have established a culture that supports such efforts”.^[^ ^31^ ^]^

### 3.3 Time resources

The magnitude of resources required to support quality improvements is often underestimated, but without adequate financial support, infrastructure, managerial skills, and dedicated time, efforts to improve quality can quickly run into difficulties.^[^ ^17^ ^]^ Time has been identified by many as a critical component of successful PQI projects.^[^ ^3^, ^17^, ^30^, ^32^, ^33^, ^34^ ^]^ Broder et al.^[^ ^3^ ^]^ advocated in their article that extra staffing is a prerequisite for successful QI projects. Extra staffing can then be used to give the required time needed to – identify and define the process or problem, collect and analyze the data, generate and prioritize solutions, and finally implement change and monitor results.^[^ ^1^ ^]^ After determination of the proper staffing and skills needed, roles and time allocation should be clearly defined.^[^ ^3^ ^]^ Kaplan et al.^[^ ^32^ ^]^ conducted a literature review of factors affecting the success of QI projects. The most frequently examined contextual factors were funding, general resources, and time. Studies that assessed time resources for QI found positive associations in 60% of the associations tested.^[^ ^35^ ^]^ Choudhery et al.^[^ ^33^ ^]^ examined radiology resident participation in PQI projects and reported that resident with dedicated time were more likely to complete a PQI and to publish their results. In a survey of 25 healthcare professionals who had recently carried out PQI projects, having limited time to perform the initiative was considered the most important barrier.^[^ ^30^ ^]^ In day‐to‐day activities, one is likely to have competing priorities and will need support to make time for QI.

## 4 CONCLUSIONS

By providing MPs dedicated time for PQI projects, management upholds its core values which include a commitment to excellence, by ingraining QI into the fabric of all clinical processes and in all aspects of the services we provide, excellence in quality and safety of clinical care, and service to our patients and customers and adherence to regulatory compliance for QI initiatives. Furthermore, it demands accountability for the dedicated time and thus more likely the success of the PQI projects. Most of the PQI projects will result in improved patient care and reduce costs in the provision of radiation therapy. Some PQI projects will generate background data for research and publications. Last, clinical MP will meet their MOC part IV requirement.

## AUTHORS CONTRIBUTIONS

All authors contributed significantly to the drafting and the final manuscript.

## CONFLICT OF INTEREST

None to declare.
